# Seasonal Changes in Nycthemeral Availability of Sympatric Temperate Mixed Forest Rodents: The Predators’ Perspective

**DOI:** 10.3390/life14010045

**Published:** 2023-12-27

**Authors:** Remo Probst, Renate Probst

**Affiliations:** Ornis—Biology Engineering Office and Research Institute, Dr. G. H. Neckheimstr. 18/3, A-9560 Feldkirchen, Austria; renate.probst@ornis-institut.at

**Keywords:** seasonal prey availability, predator-prey activity patterns, diurnality, bank vole, *Clethrionomys glareolus*, *Apodemus* mice, temperate mixed forest

## Abstract

(1) Background: Bank voles (*Clethrionomys glareolus*) and *Apodemus* mice are of exceptional importance as prey for predators in temperate mixed forests. We hypothesized that overall prey availability would increase linearly with prey frequency, and that the daylight hours, which are considered particularly dangerous, would be used only during seasonal rodent population peaks and only in the twilight hours. (2) Methods: We conducted a two-year camera-trapping study in an inner alpine mixed forest and collected 19,138 1 min videos in 215 camera-trap nights. Prey availability was defined as the pseudo-replication-limited maximum number of the respective rodent taxon per 30 min period, summed per season. (3) Results: Overall prey availability increased with frequency, i.e., the maximum number of rodent individuals per camera-trap night. Seasonally, *Apodemus* mice were particularly available to predators in the summer and bank voles in the autumn after a tree mast year. In both cases, this was accompanied by a significant increase in diurnal availability. During the population peak of *Apodemus* mice, the nocturnal availability of bank voles decreased without a concurrent increase in absolute diurnal availability, even though the significant relative shift to diurnal activity superficially suggested this. Bank voles were active throughout the day, while *Apodemus* mice were nocturnal and (rarely) crepuscular. (4) Conclusions: Availability of rodents to predators, especially during daylight hours, was mainly dependent on their tree mast-induced increased frequencies. Bank voles likewise responded strongly to interspecific competition with the larger and aggressive *Apodemus* mice, which negatively affected availability to predators. At our seasonal level of evaluation, we conclude that nycthemeral availability of forest-dwelling rodents to generalist predators of temperate mixed forests is predominantly driven by bottom-up mechanisms.

## 1. Introduction

In the arms race against predators, prey have evolved powerful defense mechanisms. Antipredator protection includes avoidance, warning signals, such as aposematism and calls, physical deterrence, and group defense [1,2,3]. In a restricted, functional sense, avoidance means staying away spatially or temporally from predators and thereby eluding detection [3]. To avoid encounters with predators, prey may alter their activity patterns according to the risk allocation hypothesis, i.e., adaptively changing temporal exposure to predation across high- and low-risk situations [4,5,6,7,8]. The majority of small, ground-dwelling mammals in the world are nocturnal, but diurnal, crepuscular, and cathemeral strategies also exist [9]. Nocturnal activity is presumably an evolutionary adaptation to an increased predation risk in the day due to the superior sensory abilities of diurnal predators [10,11,12,13]. 

Most of the time, however, there is a trade-off between activities like foraging, mating, intraguild interaction, and antipredator behavior [14,15], and multiple predator communities may increase the temporal overlap with prey [16,17]. Therefore, it is not possible for prey species, such as temperate mixed forest-dwelling rodents like *Apodemus* spp. mice (hereafter referred to as *Apodemus* mice) and bank voles (*Clethrionomys glareolus*), formerly known as *Myodes glareolus* [18], to completely avoid exposure to predators or to be active only at night [19,20,21,22]. In fact, mice of the genus *Apodemus* are predominantly nocturnal, whereas bank voles exhibit a more flexible activity pattern [23,24]. Factors influencing (increased) diurnal activity in these small mammals can be diverse and include abiotic factors such as temperature and precipitation [25,26,27,28], as well as biotic factors such as nutrition, intraspecific organization, and intraguild competition [29,30,31,32,33]. 

From the perspective of a wide range of predators, *Apodemus* mice and bank voles are important prey, and a seasonally increased prey availability is of particular interest with regard to survival and reproduction [34,35]. In inner alpine mixed forests such as our study area, this includes diurnal predators such as common buzzard (*Buteo buteo*, [36]), nocturnal predators such as tawny owl (*Strix aluco*, [37]), stone marten (*Martes foina*, [38]) and European polecat (*Mustela putorius*, [39]), as well as nycthemeral more flexible predators such as red fox (*Vulpes vulpes*, [40]), pine marten (*Martes martes*, [41]) and domestic cat (*Felis catus*, [42]). 

Our dataset covers two years of camera-trapping. We define availability as the probability that prey will be accessible to above ground hunting predators [43]. As an approximation of the overall availability of prey, the pseudo-replication-limited maximum numbers of *Apodemus* mice and bank voles of the 48 30-min periods per day were added up, separately in each of the eight seasons investigated. The highest prey frequency per camera trap-night, i.e., the maximum number of prey individuals in any of the 48 given 30-min periods per day, served as an approximation of prey abundance. We address questions related to seasonal availability of *Apodemus* mice and bank voles to their predators: 1. At first, we predicted that a higher frequency of forest rodents would lead to a proportionally increased availability, i.e., daily overall and seasonally enhanced access to prey (Prediction 1). 2. In the next step, we separated overall prey availability into different components, namely diurnal and nocturnal availability, and the relative ratio between these two. Because diurnal activity is considered particularly dangerous, we predicted that increased diurnal and in parallel nocturnal availability would only occur at high prey frequencies. Increased diurnal availability would result from an expansion of nocturnal activity, but not from a mere shift of availability into the day (Prediction 2). 3. Finally, we predicted that increased diurnal availability would result in greater use of the crepuscular margins of the day rather than mid-day. If ecologically necessary but dangerous, the activity of small forest-dwelling rodents should extend just into the light day, but not into the brightest hours (Prediction 3). 

## 2. Materials and Methods 

### 2.1. Study Site

The study was conducted on the inner alpine mountain range of the Ossiacher Tauern (46,692° N; 14,067° E) in the province of Carinthia, southern Austria. The mixed forest is situated at 550 m a. s. l. and dominated by Norway spruce (*Picea abies*), but with an admixture of European beech (*Fagus sylvatica*), limes (*Tilia platyphyllos* and *T. cordata*), sycamore (*Acer pseudoplatanus*), European ash (*Fraxinus excelsior*), European hazelnut (*Corylus avellana*), and fir (*Abies alba*). The largely closed and steep forest area consists mostly of weak tree wood, although some big “achievers” occur. In the two years of the camera trap study, mean annual temperature was 8.88 °C and 9.39 °C and the total annual precipitation was 1159 mm and 749 mm, respectively (https://data.hub.zamg.ac.at, accessed on 1 September 2023).

### 2.2. Taxonomic Identification

Bank voles can be identified up to the species level in appropriate videos; for *Apodemus* mice, this is only possible to genus level [23]. In our study area, we expect the yellow-necked mouse (*Apodemus flavicollis*) and the wood mouse (*A. sylvaticus*) to occur, while the alpine mouse (*A. alpicola*) is missing to current knowledge [44,45]. For our research question, the identification problem with the *Apodemus* mice is of minor importance, because both species under consideration are basically nocturnal [27,46].

### 2.3. Data Collection

The study was conducted from September 2020 to September 2022. The study area had a size of 4.8 ha (minimum convex polygon calculated using QGIS 3.18.0) and a random allocation layout of camera traps with 83 recording points was conducted [47]. We used Wild-Vision Full HD 5.0 camera traps with a Black-LED flash. Trigger speed was lower than 1 s and the passive infrared sensor (PIR) was designed for high sensitivity. We recorded 1 min videos in HD resolution of 1280 × 720 pixels [48]. Multiple individuals of the target species were only counted when they were simultaneously identified in a single video. 

*Apodemus* mice and bank voles were attracted with unpeeled, black-and-white sunflower seeds [24] in a quantity of 0.5 kg per camera trap [49], to be able to feed a potentially high number of small forest-dwelling rodents at population peaks and thereby increasing the sample size [50]. To ensure the spatiotemporal independence of data [51,52], we set up camera traps every 12.07 ± 6.32 sd days and in a distance of 58.94 ± 42.47 sd m, corrected for the slope. Analyses were limited to the first 24 recording h per camera trap (hereafter called a camera trap-night) and recording points were not allowed to be reused in the subsequent trial. 

### 2.4. Definitions of Terms and Data Treatment

- Mast year/non-mast year: Within the two years of study, the first was characterized by an extreme seed mast, the second by a nil crop, hereafter referred to as mast year and non-mast year, respectively [33,53].
- Seasons: Meteorological rather than astronomical seasons were used for this evaluation because they are based on the ecologically important annual temperature cycle.
- Rodent frequencies: To achieve a high level of independence of the data, the video recordings per camera trap and trap-night were divided into 48 periods with a length of 30 min. Only the single recording with the highest number of *Apodemus* mice or bank voles was used per individual 30-min period [54].
- Division day/night: Sunrise or sunset represents the boundary between day and night for the evaluated 30-min periods. The two twilight periods during a calendar day were assigned to day or night according to the higher number of corresponding minutes [33].
- Availabilities of above ground active *Apodemus* mice and bank voles to predators: (a) overall: total daily availability, diurnal and nocturnal availabilities are summarized, (b) absolute diurnal: availability exclusively in the 30-min periods of the light day, (c) relative diurnal: proportion of diurnal availability out of the overall availability (%-value), and (d): absolute nocturnal: availability exclusively in the 30-min periods of the night.

### 2.5. Data Analyses

Data were analysed using R 4.3.0 [55]. Relationships between metric and non-normally distributed variables were assessed by means of Spearman correlations. Differences between two groups regarding non-normally distributed data were examined using the non-parametric Mann–Whitney U test. In addition, multiple linear regressions were applied to analyse metric data. Loess-smoothing was used to graphically depict the progression of metric data over time. The statistical significance threshold for all analyses was set at *p* < 0.05.

## 3. Results

### 3.1. Overall Availability 

**Figure 1:**
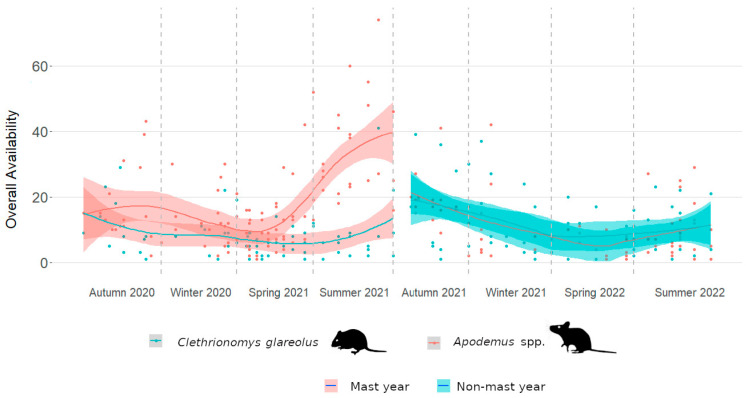
Seasonal overall availability of forest-dwelling rodents to their predators in an inner alpine study site. Significantly increased availability correlated with high frequencies of *Apodemus* mice and bank voles about one year after tree seed masting. The peak of availability occurred in the summer of 2021 for *Apodemus* mice, while it was less pronounced in autumn 2021 for bank voles. Each point represents one camera trap.

In this study, we analyzed 19,138 1 min videos collected in 215 camera-trap nights. The assessment of overall availability revealed a strong positive correlation with the maximum number of individuals per camera-trap night for both *Apodemus* mice (Spearman’s ρ: r = 0.618, *p* < 0.001) and bank voles (Spearman’s ρ: r = 0.856, *p* < 0.001). The visual impression from Figure 1, indicating that *Apodemus* mice are particularly available to predators in the summer and bank voles in the autumn after a tree mast year, is further supported by the results of the multiple linear regressions. In comparison to autumn 2020, *Apodemus* mice displayed significantly increased availability in summer 2021, followed by significant reductions in the subsequent spring and summer (Table 1). Bank voles also experienced a significant increase in availability, though not until the decline of *Apodemus* mice and the associated increased frequency in autumn 2021 (Table 2). 

### 3.2. Nycthemeral Availability 

#### 3.2.1. Absolute and Relative Diurnal Availability 

**Figure 2:**
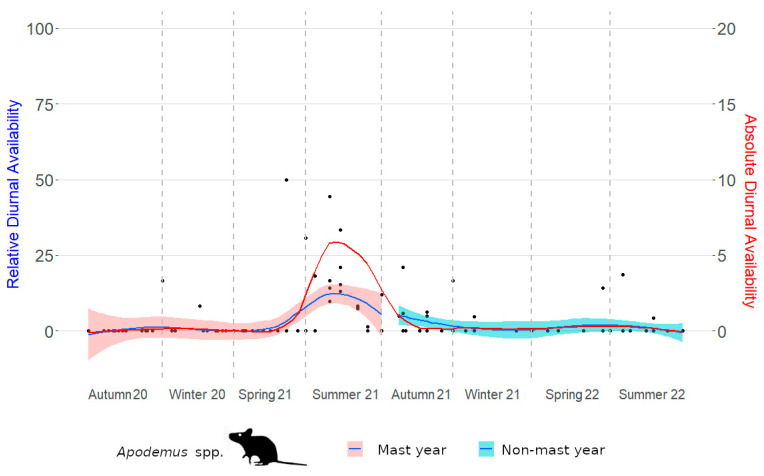
Absolute and relative diurnal availability of *Apodemus* mice in an inner alpine study site. Both availabilities increased in parallel and significantly in summer 2021. Each point represents one camera trap.

**Figure 3:**
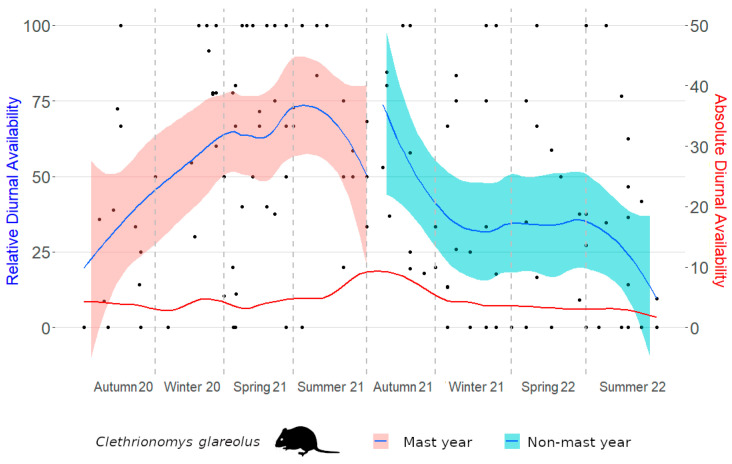
Absolute and relative diurnal availability of bank voles in an inner alpine study site. In contrast to *Apodemus* mice, absolute diurnal availability increased significantly only in autumn 2021. Bank voles were relatively, but not absolutely, more available to their predators during daylight hours from winter 2020/2021 into summer 2021. Each point represents one camera trap.

Absolute diurnal and relative diurnal availabilities are depicted (Figure 2 and Figure 3). In *Apodemus* mice, absolute diurnal availability was significantly increased only in summer 2021 (Table 3), and this equally applied to relative diurnal availability (Table 4). In bank voles, the peak of absolute diurnal availability shifted to autumn 2021 (Table 5). However, in contrast to *Apodemus* mice, this was not accompanied by increased relative diurnal availability. In fact, bank voles were relatively more available during the day from winter 2020/2021 to summer 2021 (Table 6) without a concurrent increase in absolute diurnal availability. 

#### 3.2.2. Absolute Nocturnal Availability 

In *Apodemus* mice, absolute nocturnal availability increased significantly in summer 2021 in parallel with absolute and relative diurnal activity (Table 7). Additionally, availability was significantly reduced in the spring and summer 2022, albeit with very low frequencies of these mice. In bank voles, absolute nocturnal availability decreased significantly in spring 2021 and additionally showed an almost significant reduction in summer 2021 (Table 8). In autumn 2021, when absolute diurnal availability increased significantly, this was not mirrored by an equivalent increase in absolute nocturnal availability. 

### 3.3. Diurnal Availability Pattern 

**Figure 4:**
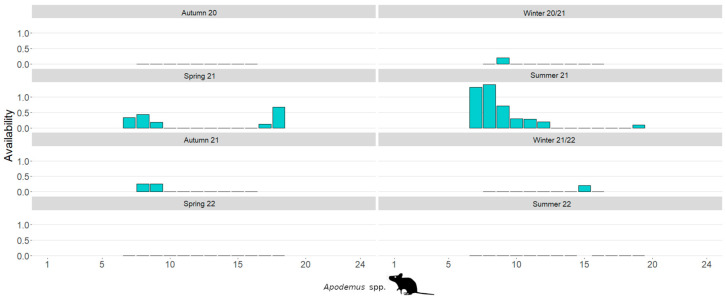
Seasonal diurnal availability of *Apodemus* mice on an inner alpine study site. Diurnal availability was an extension of nocturnal availability, primarily occurring at the edges of the light day and coinciding almost exclusively with the time of population maximum.

**Figure 5:**
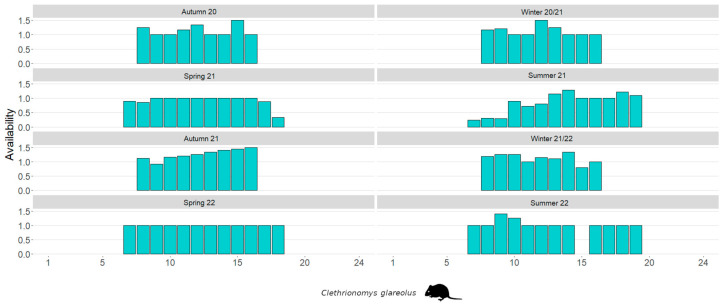
Seasonal diurnal availability of bank voles in an inner alpine study site. Availability is given for the entire light day.

The diurnal availability patterns, separated by season, are illustrated in Figure 4 for *Apodemus* mice and in Figure 5 for bank voles. Based on the average maximum frequency per 30-min period, the availability of *Apodemus* mice significantly decreases in the hours around midday (Spearman’s ρ: r = −0.160, *p* < 0.001). This is not the case with bank voles, as their availability actually increases around midday (Spearman’s ρ: r = 0.200, *p* < 0.001). 

## 4. Discussion

Diel activity pattern of prey, such as bank voles and *Apodemus* mice, is thought to be influenced by a trade-off between physiological needs and the reduction of predation risk through spatiotemporal avoidance [4,5]. This paper examines the seasonal availability of these sympatric, forest-dwelling rodents to generalist predators in an inner alpine mixed forest, with particular emphasis on the risks associated with diurnal activity [56]. We discuss the nycthemeral availability pattern in terms of intraspecific and intraguild bottom-up mechanisms (changes in prey frequency and concurrence among prey). 

Only *Apodemus* mice conformed to our predictions. When they were particularly frequent during a population peak induced by a tree mast, they were more available to predators both at night and during the day. Their heightened diurnal activity during this season was an extension of their nocturnal activity rather than a shift to daytime. This markedly increased availability of *Apodemus* mice during the summer of 2021 was undoubtedly advantageous in terms of nutrition for both territorial predators (e.g., energy-demanding, post-breeding molt [57]) and dispersing predators [39,58]. However, after the population collapsed in the autumn of 2021, availability significantly decreased also at night from spring to summer 2022, thus possibly negatively affecting the predators’ reproductive phase in the following year [59]. 

Therefore, the availability to predators in this genus was strongly influenced by intraspecific and density-dependent mechanisms. *Apodemus* mice possess several traits that enable them to evade diurnal activity. They dominate over competitors such as bank voles [60], consume and store high-energy food [31], and can slow down their metabolism under certain circumstances [61,62]. In addition, sharpened sensory capabilities make them well-equipped to survive nocturnal predation attempts [23]. The increased diurnal availability cannot be attributed solely to the shorter nights in summer because it was not observed at low population densities in summer 2022 and availability was primarily concentrated to the twilight hours of the day. In conclusion, for predators, this translates to particularly high availability of *Apodemus* mice during population peaks, both at night [37,63,64] and even during the day [65]. 

Bank voles displayed considerably more varied responses in their chronoecology and barely met our predictions. Particularly in the spring and summer of 2021, both overall and absolute nocturnal activity was reduced, and availability relatively shifted into the daytime. This resulted in a decrease in availability to nocturnal predators without a corresponding increase in absolute diurnal availability, despite the calculation of the relative value superficially suggesting otherwise [66]. This inverse activity pattern closely coincided with the population maximum of the dominant *Apodemus* mice and can be interpreted as a strategy to avoid intraguild competition [33]. It was not until bank voles themselves reached a small population peak in the autumn of 2021 that absolute diurnal availability increased. By this time, the population of *Apodemus* mice had already collapsed, suggesting an intraspecific and density-driven effect as well. It can be surmised that the competition-induced reduction in overall availability in spring was facilitated by the availability of high-energy, less foraging time-consuming food obtainable during this season. However, this reduction possibly had a negative impact on population growth and may have shifted the frequency peak further into the autumn. 

Bank voles were thus much more likely than *Apodemus* mice to exhibit diurnal activity, and this was not restricted to the twilight hours of the day. The increase in absolute diurnal availability observed in the autumn of 2021 was not accompanied by a parallel increase in absolute nocturnal availability either. Therefore, bank voles must have evolved effective antipredator strategies to keep diurnal mortality low or to derive other advantages from diurnal activity. Some potential benefits include reduced daily energy expenditure, as predicted by the circadian thermo-energetics hypothesis [61], as well as advantages in foraging and digestion [31,67]. Individual personality differences in bank voles may also account for some of the diurnal variation in risky behaviors such as foraging [68,69]. From the predators’ perspective, bank voles are known for their use of cover-rich microhabitats [70,71], making them relatively difficult to detect and capture, even during the day. Extensive pellet analyses in Central Europe involving a variety of diurnal and nocturnal predators, both avian and mammalian, have shown that the bank vole is notably underrepresented relative to its abundance [72]. The same is true for the boreonemoral region, where mice of the genus *Microtus* are preferred by various owl species [73].

At our seasonal day-night evaluation level, we hypothesize a bottom-up controlled predator-prey system. Seasonal and diurnal variations in prey availability were strongly influenced by intraspecific, density-dependent organization in *Apodemus* mice and bank voles as well. Furthermore, multiple regression analysis indicated a linear relationship between frequency and availability. Non-linear or exponential correlations were ruled out through examination of scatter plots. Bank voles were further influenced by competitive mechanisms within the guild of forest-dwelling rodents. As expected, when both *Apodemus* mice and bank voles were more frequent, their availability increased overall [74], but especially during the day. Surprisingly, at the peak of *Apodemus* mice frequency, interspecific competition significantly reduced bank vole availability both overall and during the night, without causing a shift in absolute availability to the daytime. 

While we were able to detect the avoidance of moonlight as an indirect cue of predation risk in our study area [33], we found no evidence indicating that the generalist predators of this temperate forest ecosystem were responsible for the seasonal changes in the overall nycthemeral activity patterns of their rodent prey. We mainly recorded nocturnal generalist predators in the non-mast year (*n* = 13; red fox, 53.8%; stone marten, 23.1%; pine marten, 7.7%; European polecat, 7.7%; tawny owl, 7.7%), i.e., at the time when nocturnal activity was prevalent in *Apodemus* mice. Moreover, there was no difference in absolute diurnal availability with respect to the detection or non-detection of nocturnal predators in bank voles as well (W = 306.50, *p* = 0.811). Therefore, it is highly unlikely that predators induced increased diurnal activity, even via indirect cues such as feces, urine, and anal gland secretions [75,76]. In the predator-prey-system we investigated, the system-stabilizing generalist predators [77] are apparently unable to induce a temporal niche switching in rodents, from primarily nocturnal activity to predominantly diurnal activity, or vice versa. We conclude that predators in our study area need to adapt their hunting patterns to match the temporal availability of prey [13]. Resident specialized predators *sensu* [78] may have a greater influence on the nycthemeral activity of their rodent prey in their ongoing “David and Goliath” arms race [21,35,79,80,81,82]. However, we never detected highly specialized vole-hunting species such as stoat (*Mustela erminea*) and least weasel (*Mustela nivalis*) during our two years of study, neither by the camera traps nor by observations during field work. 

We were only able to identify bank voles up to species and *Apodemus* mice up to genus level. Regarding behavioral choices made in response to predation risk, especially the important decisions about when, where, and what to feed [83], we primarily highlighted the first aspect. We had no data on the specific diets of our prey taxa, and our camera trap locations shared a high degree of habitat similarity [33]. Nevertheless, we did observe a “thigmotaxis parameter” (% of cover with lying deadwood, snags, and rocks in a 10 m radius of the camera trap) targeting the bank vole’s need for cover, which showed a negative correlation with overall availability (Spearman’s ρ: r = −0.217, *p* = 0.011). This suggested that with increasing frequency, bank voles possibly had to leave the sheltered cover more frequently. Conversely, there was a positive correlation for *Apodemus* mice, which are generally more socially tolerant (Spearman’s ρ: r = 0.865, *p* < 0.001). For a more comprehensive understanding of species-, sex-, age-, nutrition-, and habitat-specific characteristics, as well as effects of diel vulnerability to predation in the future, it would be promising to combine the camera trap survey with live-trapping [84,85,86], diet tracing [31,87,88,89,90], and further related aspects [69,91,92,93,94,95,96]. This approach would also enable a more precise measurement of the crucial parameter of availability [97]. 

We are, nevertheless, convinced to have made a methodological and subject-related contribution to the understanding of seasonal changes in nycthemeral availability of a temperate mixed forest-dwelling rodent community from the predators’ perspective:

1. We used the video function of camera traps, which is better suited than the photo function for determining the frequency (and behavior) of small mammals.
2. Camera traps reduce the need for handling and thus minimize disturbance of the target organisms. We were able to accurately determine the nycthemeral activity of *Apodemus* mice and bank voles because we completely avoided manipulations during the twilight hours.
3. We conducted a two-year, year-round study, allowing us to cover all seasons with a large sample of videos.
4. The tree mast/nil crop-induced outbreak-crash pattern in *Apodemus* mice in our study provided us with a quasi-experimental situation to measure the influence of the dominant competitor.
5. Overall above ground availability to predators (summed maxima of prey individuals of the 48 30-min periods/camera trap-night) increased linearly with frequency (maximum number of prey individuals/camera trap-night) in *Apodemus* mice as well as in bank voles.
6. Seasonally, *Apodemus* mice were only available to diurnal predators at times of high population densities; in bank voles, diurnal activity increased at a (small) population peak.
7. We were able to show that the commonly used relative measure of nycthemeral activity in prey animals can lead to misconceptions about availability to predators. During the population peak of *Apodemus* mice, bank voles were diurnally active for up to three quarters of their activity time, without changing the absolute duration and, thus, their availability in daylight hours to predators.
8. Our study suggests that in a temperate mixed forest, prey availability is bottom-up controlled. This mainly depends on intraspecific, density-dependent population phenomena and is also influenced by intra-guild competition with *Apodemus* mice in the case of the bank vole. We found no evidence for control of this forest predator-prey system by the generalist, predominantly non-migratory predators.

## Figures and Tables

**Table 1:** Seasonal overall availability of *Apodemus* mice. Adjusted R^2^ = 0.356.

Season	Estimate	Standard Error	t-Values	*p*-Values
(Intercept)	17.118	2.517	6.802	<0.001
Winter 2020/2021	−3.832	3.385	−1.132	0.259
Spring 2021	−4.318	3.004	−1.437	0.153
Summer 2021	17.692	3.385	5.226	<0.001
Autumn 2021	0.588	3.559	0.165	0.869
Winter 2021/2022	−5.918	4.135	−1.431	0.154
Spring 2022	−12.027	4.015	−2.995	0.003
Summer 2022	−6.858	3.213	−2.135	0.034

**Table 2:** Seasonal overall availability of bank voles. Adjusted R^2^ = 0.083.

Season	Estimate	Standard Error	t-Values	*p*-Values
(Intercept)	10.308	2.235	4.611	<0.001
Winter 2020/2021	−1.308	3.161	−0.414	0.680
Spring 2021	−4.234	2.721	−1.556	0.122
Summer 2021	−1.808	3.009	−0.601	0.549
Autumn 2021	6.923	3.161	2.190	0.030
Winter 2021/2022	1.392	2.871	0.485	0.629
Spring 2022	−1.955	2.970	−0.658	0.512
Summer 2022	0.339	2.970	0.114	0.909

**Table 3:** Absolute diurnal availability of *Apodemus* mice. Adjusted R^2^ = 0.216.

Season	Estimate	Standard Error	t-Values	*p*-Values
(Intercept)	0.059	0.603	0.098	0.922
Winter 2020/2021	−0.011	0.811	−0.014	0.989
Spring 2021	0.416	0.720	0.578	0.564
Summer 2021	4.370	0.811	5.388	<0.001
Autumn 2021	0.588	0.853	0.690	0.491
Winter 2021/2022	0.141	0.991	0.142	0.887
Spring 2022	0.032	0.962	0.033	0.973
Summer 2022	0.163	0.770	0.212	0.832

**Table 4:** Relative diurnal availability of *Apodemus* mice. Adjusted R^2^ = 0.186.

Season	Estimate	Standard Error	t-Values	*p*-Values
(Intercept)	0.980	1.731	0.566	0.572
Winter 2020/2021	−0.584	2.329	−0.251	0.802
Spring 2021	1.039	2.067	0.503	0.616
Summer 2021	11.231	2.329	4.822	<0.001
Autumn 2021	2.533	2.448	1.035	0.302
Winter 2021/2022	−0.504	2.845	−0.177	0.860
Spring 2022	0.318	2.762	0.115	0.908
Summer 2022	−0.133	2.210	−0.060	0.952

**Table 5:** Absolute diurnal availability of bank voles. Adjusted R^2^ = 0.037.

Season	Estimate	Standard Error	t-Values	*p*-Values
(Intercept)	4.000	1.384	2.891	0.005
Winter 2020/2021	1.000	1.957	0.511	0.610
Spring 2021	−0.222	1.684	−0.132	0.895
Summer 2021	1.813	1.863	0.973	0.332
Autumn 2021	4.308	1.957	2.201	0.029
Winter 2021/2022	−0.300	1.777	−0.169	0.866
Spring 2022	−0.647	1.838	−0.352	0.725
Summer 2022	−0.882	1.838	−0.480	0.632

**Table 6:** Relative diurnal availability of bank voles. Adjusted R^2^ = 0.153.

Season	Estimate	Standard Error	t-Values	*p*-Values
(Intercept)	34.231	9.349	3.661	<0.001
Winter 2020/2021	29.582	13.222	2.237	0.027
Spring 2021	28.444	11.380	2.500	0.014
Summer 2021	33.793	12.587	2.685	0.008
Autumn 2021	21.764	13.222	1.646	0.102
Winter 2021/2022	−2.793	12.009	−0.233	0.816
Spring 2022	1.859	12.420	0.150	0.881
Summer 2022	−9.392	12.420	−0.756	0.451

**Table 7:** Absolute nocturnal availability of *Apodemus* mice. Adjusted R^2^ = 0.301.

Season	Estimate	Standard Error	t-Values	*p*-Values
(Intercept)	17.059	2.369	7.200	<0.001
Winter 2020/2021	−3.821	3.187	−1.199	0.232
Spring 2021	−4.734	2.828	−1.674	0.096
Summer 2021	13.322	3.187	4.180	<0.001
Autumn 2021	0.000	3.351	0.000	1.000
Winter 2021/2022	−6.059	3.893	−1.556	0.122
Spring 2022	−12.059	3.780	−3.190	0.002
Summer 2022	−7.022	3.025	−2.321	0.022

**Table 8:** Absolute nocturnal availability of bank voles. Adjusted R^2^ = 0.116.

Season	Estimate	Standard Error	t-Values	*p*-Values
(Intercept)	6.308	1.567	4.026	<0.001
Winter 2020/2021	−2.308	2.216	−1.041	0.300
Spring 2021	−4.011	1.907	−2.103	0.037
Summer 2021	−3.620	2.109	−1.716	0.089
Autumn 2021	2.615	2.216	1.180	0.240
Winter 2021/2022	1.692	2.013	0.841	0.402
Spring 2022	−1.308	2.081	−0.628	0.531
Summer 2022	1.222	2.081	0.587	0.558

## Data Availability

All data, tables, and figures are original. Details on data availability can be obtained from the corresponding author upon reasonable request.
